# Determination of Admission Criteria for Global Public Health Master at the University of Georgia

**DOI:** 10.1093/eurpub/ckac131.384

**Published:** 2022-10-25

**Authors:** T Lobjanidze, N Skhvitaridze, K Antia, M Lobjanidze, M Nonikashvili, G Shakarishvili

**Affiliations:** School of Health Sciences and Public Health, University of Georgia, Tbilisi, Georgia

## Abstract

**Background:**

Recent events in the world, wars, pandemics, have once again raised the issue of the need for competent human resources in global public health (GPH). In Georgia, there are master’s degree programs in public health where global health is mostly offered as an elective or core course of the curriculum. University of Georgia (UG) is involved in the ERASMUS + project in GPH (BACE). UG initiated to create new GPH Master Program, which will be response towards local, regional and international challenges and demand.

**Objective:**

Main objective of developing GPH program was to address and meet national and international global health education needs and challenges. The aim of the study was to identify evidence-based prerequisites for the MPH in GPH (UG). We conducting document review based on the GPH competency model (GPHCM) and evaluated: 1) GPH & MPH programs of the top 20 world university ranking (THE/2022); 2) CUGH associated programs; 3) Georgian MPH programs; 4) Georgian education documents (such as Georgian National Qualifications Framework (GNQF)); 5) Main directions of career development in global health. In addition, interviews with national experts were conducted (in education, employment, program leaders) using the Delph method.

**Results:**

Study found that depending on the scope and content of the program, prerequisites for obtaining MPH in GPH varies. In addition to English competence and a high GPA, working or research experience or interviews or additional exams or other are required. In Georgia, there is no adopted GPHCM, but GNQF allows the establishment of MPH in GPH. UG new Master program (120 ECTS) will include 99 ECTS core (including research practicum & thesis) and 21 ECTS elective courses based on 6 domains of GPHCM. The program will mostly focus on population health management at a global level.

**Conclusions:**

Study findings suggest any undergraduates with appropriate conditions could be eligible for admission to the UG (MPH in GPH).

**Key messages:**

• UG admission prerequisites for Health Science undergraduates: National entrance exam, English proficiency B2, working experience and motivation letter.

• UG additionally admission prerequisites for Health Science undergraduates: basic knowledge epidemiology and medical terminology (if credits available or test).

